# Human-Centered Design Study: Enhancing the Usability of a Mobile Phone App in an Integrated Falls Risk Detection System for Use by Older Adult Users

**DOI:** 10.2196/mhealth.7046

**Published:** 2017-05-30

**Authors:** Richard Harte, Leo R Quinlan, Liam Glynn, Alejandro Rodríguez-Molinero, Paul MA Baker, Thomas Scharf, Gearóid ÓLaighin

**Affiliations:** ^1^ NUI Galway Electrical and Electronic Engineering School of Engineering & Informatics Galway Ireland; ^2^ CÚRAM SFI Centre for Research in Medical Devices Human Movement Laboratory NUI Galway Galway Ireland; ^3^ NUI Galway Physiology School of Medicine Galway Ireland; ^4^ NUI Galway General Practice School of Medicine Galway Ireland; ^5^ Consorci Sanitari del Garraf Çlinical Research Unit Vilanova i la Geltrú Barcelona Spain; ^6^ CACP Center for Advanced Communications Policy Georgia Institute of Technology North Avenue NW GA 30332 Atlanta, GA United States; ^7^ NUI Galway Irish Centre for Social Gerontology Institute for Lifecourse and Society Galway Ireland

**Keywords:** human-centered design, user-centered design, human-computer interface, human factors engineering, eHealth, engineering psychology, mHealth

## Abstract

**Background:**

Design processes such as human-centered design (HCD), which involve the end user throughout the product development and testing process, can be crucial in ensuring that the product meets the needs and capabilities of the user, particularly in terms of safety and user experience. The structured and iterative nature of HCD can often conflict with the necessary rapid product development life-cycles associated with the competitive connected health industry.

**Objective:**

The aim of this study was to apply a structured HCD methodology to the development of a smartphone app that was to be used within a connected health fall risk detection system. Our methodology utilizes so called discount usability engineering techniques to minimize the burden on resources during development and maintain a rapid pace of development. This study will provide prospective designers a detailed description of the application of a HCD methodology.

**Methods:**

A 3-phase methodology was applied. In the first phase, a descriptive “use case” was developed by the system designers and analyzed by both expert stakeholders and end users. The use case described the use of the app and how various actors would interact with it and in what context. A working app prototype and a user manual were then developed based on this feedback and were subjected to a rigorous usability inspection. Further changes were made both to the interface and support documentation. The now advanced prototype was exposed to user testing by end users where further design recommendations were made.

**Results:**

With combined expert and end-user analysis of a comprehensive use case having originally identified 21 problems with the system interface, we have only seen and observed 3 of these problems in user testing, implying that 18 problems were eliminated between phase 1 and 3. Satisfactory ratings were obtained during validation testing by both experts and end users, and final testing by users shows the system requires low mental, physical, and temporal demands according to the NASA Task Load Index (NASA-TLX).

**Conclusions:**

From our observation of older adults’ interactions with smartphone interfaces, there were some recurring themes. Clear and relevant feedback as the user attempts to complete a task is critical. Feedback should include pop-ups, sound tones, color or texture changes, or icon changes to indicate that a function has been completed successfully, such as for the connection sequence. For text feedback, clear and unambiguous language should be used so as not to create anxiety, particularly when it comes to saving data. Warning tones or symbols, such as caution symbols or shrill tones, should only be used if absolutely necessary. Our HCD methodology, designed and implemented based on the principles of the International Standard Organizaton (ISO) 9241-210 standard, produced a functional app interface within a short production cycle, which is now suitable for use by older adults in long term clinical trials.

## Introduction

**Figure 1:**
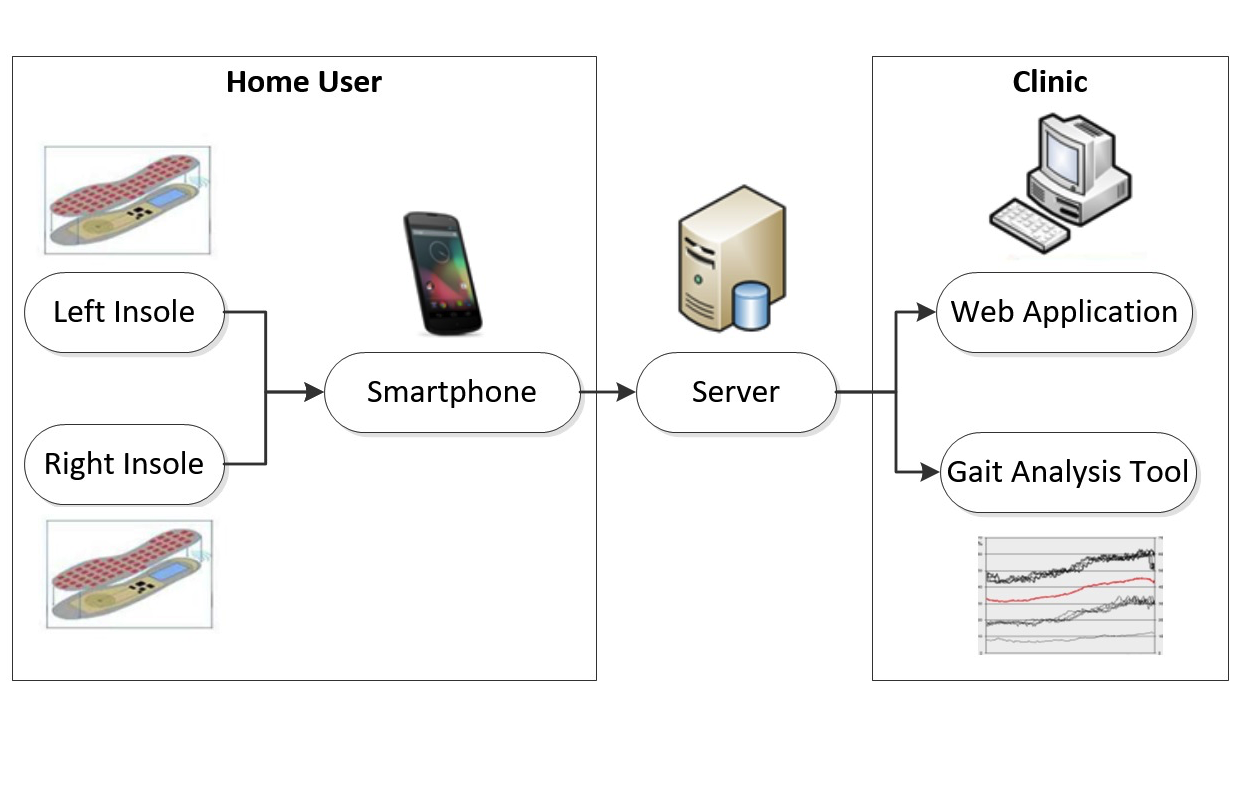
The wireless insole for independent and safe elderly living (WIISEL) system.

Utilizing a human-centered design (HCD) approach, such as that outlined in the International Standards Organization (ISO) 9241-210 [1], during the design of connected health devices ensures that the needs and requirements of the user are taken into consideration throughout the design process. HCD is a multi-stage process that allows for various iterations of a design and subsequent update to the requirements. The importance of involving end users in the design process of health products is recognized, and different approaches have been demonstrated in literature [2-8]. In this paper, we present the implementation of a structured HCD methodology, based on ISO-9241-210, which utilized standard, established techniques to assess and develop the usability and human factors of a smartphone interface with the full involvement of end users and stakeholders. The smartphone interface that was developed and tested is a component of the wireless insole for independent and safe elderly living (WIISEL) system, a system designed to continuously assess fall risk by measuring gait and balance parameters associated with fall risk. The system is also designed to detect falls. The architecture of the system is illustrated in Figure 1. It is proposed that the system can be worn at home by a user for a period of time in order to identify specific gait and balance patterns that may be affecting a user’s fall risk. The system is targeted at older adults who represent a high fall risk group. The system consists of a pair of instrumented insoles and a smartphone that are worn by the user. Data collected by embedded sensors in the insoles are sent to the smartphone, where they are then uploaded to a server in a clinic for processing and analysis. The smartphone represents a major interface in the system as this is how the home user will primarily interact with the WIISEL system with the WIISEL app, allowing the user to check the system status, sync with the insoles, send data to their local clinic, and monitor their daily activity.

The acquisition and comprehension of information from interfaces can become more difficult as a person progresses into older age. Interfaces in electronic health or medical apps can often be crowded with text and characters, have poor contrast, contain many different colors, and may not present adequate haptic or audio feedback. In terms of visual perception, age-related declines in acuity, contrast sensitivity, and ability to discriminate colors can affect reading rates, character and symbol identification, and button striking accuracy, even with optimal corrections in place [9]. Age-related cognitive decline in domains such as reasoning and memory can affect the ability of the user to comprehend the process they are perceiving on the interface [10]. Deterioration of psychomotor processes such as fine motor control and dexterity can cause problems for users attempting to interact with the physical hardware of the interface [4]. Typically between the ages of 60 and 80 years, individuals can expect up to a 50% decline in visual acuity (particularly in low luminance, low contrast, and glare environments), a reduction in hearing sensitivity by 20dBs, a 14% decline in short-term memory, and a 30% decline in power grip strength, all of which impact how one interacts with computer interfaces [11]. In addition to these physical considerations, older adults can also present a complex user group in terms of attitude toward and previous experience with technology [11].

## Methods

A 3-stage HCD methodology was utilized to enhance the usability and user experience of the smartphone app. This methodology was previously described by Harte et al [12].

### Phase 1

#### Use Case Development

The use case document outlined 7 scenarios where the user must directly interact with the smartphone interface. These scenarios were (1) the user logs in to the app, (2) the user syncs the app to the insoles, (3) the user checks the system status, (4) the user uploads the data, (5) the user minimizes the app, (6) the user resets the app, and (7) the user triggers a fall alarm. The use case, which was termed paper prototype version 1, was exposed to 2 groups of stakeholders in the form of structured analysis in order to illicit their feedback [7,13,14].

#### Expert Use Case Analysis

A total of 10 experts were selected to analyze the use case. The experts were selected from National University of Ireland (NUI), Galway based on their involvement with work related to the use of technology by older adults. We sought multi-disciplinary perspectives, as advised in ISO-92410, and therefore the group consisted of nurses, occupational therapists, physiotherapists, general practitioners, gerontologists, and engineers. The precise expertise of each expert, as well as a self-reported measure of their knowledge of (1) usability and human factors and how it can influence technology use; (2) the end user, their capabilities, and their preferences for technology; and (3) connected health devices that are used in the home can be found in Table 1.

In addition to filling out the Likert statements at the end of each scenario, the expert was instructed to engage in a think-aloud protocol as they walked through each scenario [15]. All feedback was captured by an audio recorder.

#### End User Representatives Use Case Analysis

A total of 12 older adults were recruited using a typical purposive sample (Inclusion: age 65+ years, community dwelling; Exclusion: profound hearing or vision loss, psychiatric morbidities, and severe neurological impairments) to analyze the use case. The same protocol and interview structure was used to expose the use case document to the older adults and was carried out in the home of the participant. Ethical approval to carry out the interviews and assessments was approved by University Hospital Galway (UHG) research ethics committee. For this analysis, we sought to measure, where applicable, the capabilities a user would call upon to successfully use an interface, so that we could be satisfied that test participants were representative of the target end-user population.

**Table 1:** Experts involved in use case analysis. Each of the experts was asked to mark out of 10 where they felt their own expertise of usability, the end user, and connected health lay.

#	Profession	Specific experience	End user knowledge	Usability knowledge	Connected health knowledge
1	Clinical researcher in general practice	Industry experience in software design. Research interests include the perception of older adults in the media and the quality of life of dementia sufferers in long stay care.	9	8	7
2	Occupational therapist	Experience in the delivery of occupational health solutions to older adults including ADL^a^ assessments, environmental risk assessments, cognitive assessments, and fall prevention strategies.	9	6	3
3	Senior lecturer in nursing	Registered general nurse with a PhD qualification in clinical nursing and has expert experience of treating older adults.	8	8	6
4	GP^b^ and senior lecturer	Research addresses chronic disease management and implementing connected health solutions for the management of chronic diseases.	9	5	7
5	GP and head of general practice department	Senior lecturer of general practice and lead researcher in clinical training or teaching practices and methods, as well as workplace learning and development.	9	6	4
6	Psychology researcher	Holds a PhD in psychology with research interest in team situation awareness in critical environments and designing instructional materials. Currently working in the area of examining lifestyle and technology factors associated with gestational diabetes mellitus.	7	8	7
7	Clinical researcher in general practice	Former practising nurse currently a masters researcher pursuing projects in connected health and telehealth solutions in rural communities.	8	6	8
8	GP and senior lecturer in general practice	HRB^c^ Cochrane Fellow currently practicing as a GP with expert experience of treating older adult patients. Research interests are in multimorbidity with a focus on connected health solutions.	10	6	8
9	IT^d^ lecturer and expert in user-centered design	IT researcher specialising in human computer interaction. Research interests heavily focused on the employment of user-centered design techniques for mobile devices.	6	8	4
10	Geriatrician and professor of geron-technology	MD specializing in geriatrics and PhD qualification in preventive medicine and public health. Has expert experience of treating older adults as well as specific research interests in epidemiology, geron-technology, and tele-health care.	10	8	8
		Average expert group knowledge of key areas.	8.4	7	6.4

^a^ADL: activities of daily living.

^b^GP: general practitioner.

^c^HRB: health research board.

^d^IT: information technology.

**Figure 2:**
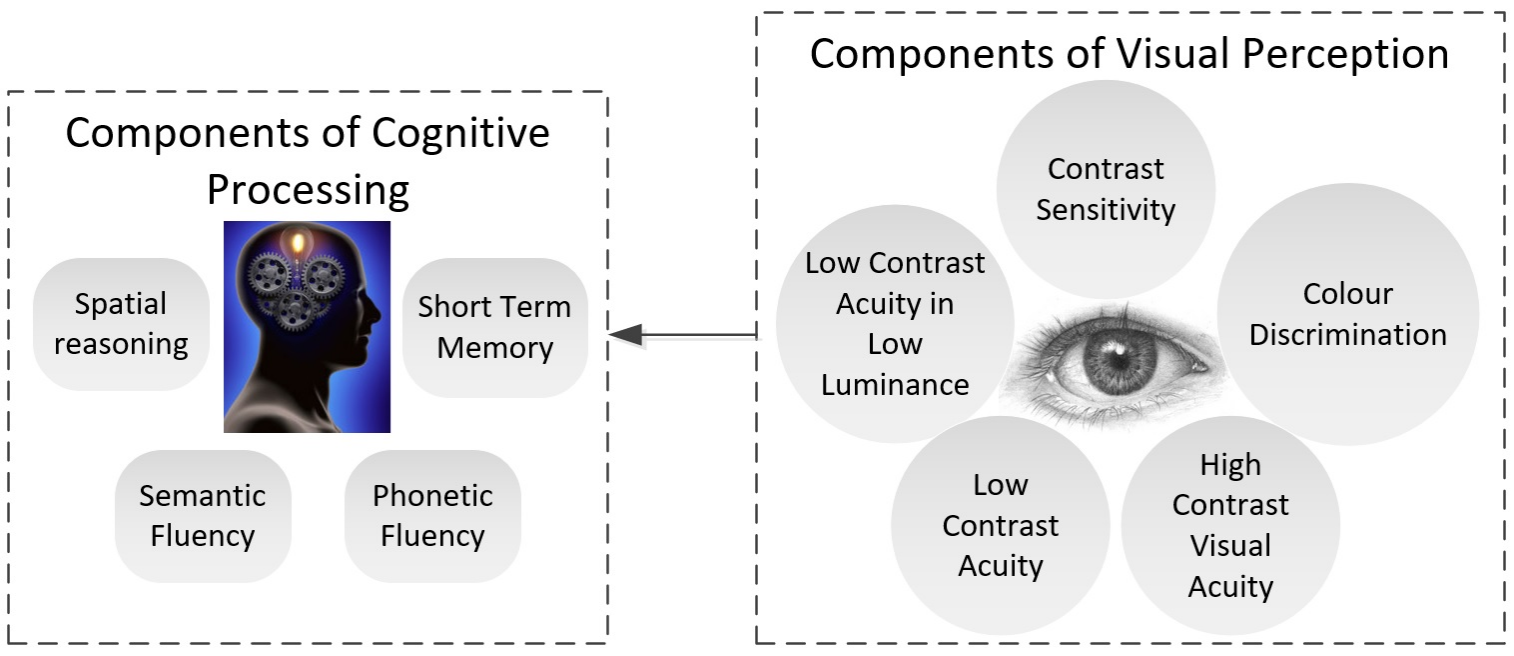
Physiological capabilities required to interact with use case.

We measured the cognitive and visual capabilities of the user and the components of the processes we measured are illustrated in Figure 2.

We used a short battery of standardized tests to measure each of the capabilities presented in Figure 2. The tests and their relevance to the analysis are listed in Table 2.

High contrast acuity (HCA) was measured using a Snellen chart at a distance of 3m. Low contrast acuity (LCA) was measured for 5% and 25% contrast using SLOAN letter charts at a distance of 3m. Standardized illumination was provided for these 2 tests using a light box from Precision Vision (precision-vision.com). Constrast sensitivity (CS) was measured using a MARS chart at a distance of 40cm, whereas low contrast acuity in low luminance (LCALL) was measured with a SKI chart at a distance of 40cm. Color discrimination (CD) was measured using a Farnsboro D-15 test. Reading acuity (RA) was measured using a Jaeger chart at a distance of 40cm. Each participant also completed 2 cognitive performance tests based on the Whitehall study [22]. Spatial reasoning was assessed using the Alice Heim 4-I (AH4-I). The AH4-I tests inductive reasoning, measuring one’s ability to identify patterns, and to infer principles and rules [24]. Short-term memory was assessed with a 20-word free recall test. Expected values of each test per age group and the actual measured can be found in Tables 3 and 4.

**Table 2:** Battery of tests.

Interactive process	Measure	Meaning and relevance
Visual perception		
	High contrast acuity	A general measure of visual capability and the ability to discern spaces between characters on a 100% contrast interface [16].
	Reading acuity	A measure of acuity when reading full words on an interface [17].
	Low contrast acuity	A general measure of visual capability and the ability to discern spaces between characters on a 5% and 25% contrast interface [18].
	Contrast sensitivity threshold	The contrast threshold at which the user can successfully identify a character [19].
	Color discrimination	Ability to discriminate colors on an interface [20].
	Low contrast acuity in low luminance	The ability to discern spaces between characters on a low contrast and poorly illuminated surface.
Cognitive processing		
	Spatial reasoning	The ability to interpret space on an interface and infer relationships between elements has been cited as a major component of website usability and software interfaces in general [9,21,22].
	Short-term memory	Memory, specifically short-term memory has been cited as an important factor in one’s ability to maintain visual attention of an interface [22,23].

**Table 3:** Average visual performance metrics measured and split by age group. The average is compared with the expected score for that age group. Data presented in each column as expected or measured

Age (years)	n (number of participants who fall into age group)	HCA^a^ (expected or measured)	RA^b^ (expected or measured)	LCA^c^ (expected or measured)	LCALL^d^ (expected or measured)		CS^e^ (expected or measured)	CD^f^
61-65	1	1/1	1/1	0.67/0.79	0.33/0.41		1.68/1.8	No defects
66-70	2	1/1	1/1	0.62/0.71	0.29/0.27		1.54/1.55	No defects
71-75	3	0.91/0.83	0.91/0.8	0.49/0.64	0.25/0.22		1.42/1.33	1 participant with very mild blue yellow confusion (tritanopia)
76-80	5	0.83/0.88	Not applicable	0.4/0.54	0.2/0.22		1.2/1.42	1 participant with very moderate blue yellow confusion (tritanopia)
81-85	1	0.76/0.66	Not applicable	0.3/0.5	0.17/0.2		0.61/0.7	No defects

^a^HCA: high contrast acuity.

^b^RA: reading acuity.

^c^LCA: low contrast acuity.

^d^LCALL: low contrast acuity in low luminance.

^e^CS: color sensitivity.

^f^CD: color discrimination.

**Table 4:** Expected scores and mean measured scores for cognitive tests for all 12 participants. The average is compared with the expected score for that age group. Data presented in each column.

Age (years)	n (number of participants who fall into age group)	Spatial reasoning (range 0-65) (expected or measured)	Short-term memory (range 0-20) (expected or measured)
61-65	1	(30-46)/30	(6.21-6.43)/8
66-70	2	(30-46)/38.5	(4.79-5.74)/6.5
71-75	3	(29.7-40)/35	(4.7-5.5)/6.3
76-80	5	(29.7-40)/29.8	(4.3-5.4)/5.9
81-85	1	(25-40)/26	(4-5.1)/4

#### Identification and Categorisation of Usability Problems

The audio feedback acquired during the analysis of the use case document by the experts and end users was “intelligently” transcribed [25] and clearly defined usability problems were extracted from the transcript. All of the problems identified by each expert, and end user were collated for each scenario. All problems were documented and illustrated in a structured usability and human factors problems report [26] and were accompanied by selected testimony from a corresponding expert or end user who elaborated on the nature of the problem for the purpose of the design team. This report was analyzed by system designers who provided potential solutions to each problem where possible.

### Phase 2

In response to the feedback from phase 1, a new paper prototype was developed (paper prototype version 2) and made available for expert inspection. A working version of the app with accompanying user manuals was also developed on a Google Nexus 5 smartphone (working prototype version 1) and made available for expert walkthrough. We returned to the original experts and carried out a 2-part usability inspection. First, the experts inspected the solutions to the problems they had identified in phase 1 using a new version of the use case (paper prototype version 2) as a guide. This use case only presented the problems that the experts identified in their original analysis and showed how the problems had been addressed. Second, they inspected the prototype app (working prototype version 1) utilizing a cognitive and contextual walkthrough methodology.

### Phase 3

The new manuals and updated interface (working prototype version 2) were exposed to the 10 older adults who had previously analyzed the use case (2 of the 12 subjects who had originally analyzed the use case were unavailable in phase 3 testing). After measuring the time taken to complete each task and the number of errors made, the after scenario questionnaire (ASQ) and the NASA Task Load Index (NASA-TLX) were administered to the participant after the task was completed. The ASQ is a Likert scale that interrogates a user’s perception of efficiency, ease of use, and satisfaction with manual support [27]. The NASA-TLX is a multi-dimensional rating procedure that provides an overall workload score based on a weighted average of ratings on 6 subscales: (1) mental demands, (2) physical demands, (3) temporal demands, (4) own performance, (5) effort, and (6) frustration [28].

## Results

This section presents the summary of results from each phase, as well as the changes made to the interface and support documentation after each phase.

### Phase 1: Use Case Analysis (Paper Prototype Version 1)

The combined expert analysis and end user analysis identified 21 problems. We have provided 13 examples of problems, which are presented in Table 5. These 13 problems were chosen for illustration because they represent unique problems, the other 8 problems were considered repetitions or derivatives of the other 13, and therefore, we felt it was not important to describe them. The problem ID number assigned to each problem was used for the remainder of the design process to allow for easier problem tracking throughout the process.

The problems from Table 5 are presented in Table 6 in order of severity rating based on the mean Likert scores assigned by the experts. The maximum individual score that was given by the 10 experts is also included to highlight the fact that some experts may have given a more severe rating than what the mean or standard deviation indicates. The heuristic category to which each problem belongs is also included.

**Table 5:** List of identified problems and which use case scenario it was identified in.

Problem ID number	Problem description (use case scenario)
1	The difference in operation between the home button and back button is not clear (user minimizes app)
2	Overall login sequence (user must log in to the app)
3	Buttons on keypad are too small for this population (user must log in to the app)
4	WIISEL^a^icon not prominent enough on app menu (user must check the system status)
5	Having to upload the data will be too hard to remember to do (uploading data by exiting app)
6	Feedback during the process is not clear or may cause anxiety (uploading data by exiting app)
7	No prompt to indicate to the user that a manual connection is now required (user must connect to the insoles)
8	Colors are too similar in places (uploading data by exiting app)
9	Feedback regarding connection status is unclear (user connects to insoles using app)
10	Homescreen information is not clear (user must check the system status)
11	Options presented are not clear (fall alarm or notification)
12	App text is too small (user must check the system status)
13	Buttons on exit screen need to be bigger (uploading data by exiting app)

^a^WIISEL: wireless insole for independent and safe elderly living.

**Table 6:** Problems uncovered by experts and rated based on mean Likert scores.

Problem ID number	Heuristic category	Severity rating (0-4) (σ)	Max severity rating given (0-4)
1	Cognitive directness	2.5 (1.2)	4
2	Consistency and compliance of task structure	2.4 (1.1)	4
3	Discernibility (button size)	2.2 (1.3)	4
4	Discernibility (icons)	2.2 (1.3)	4
5	Consistency and compliance of task structure	2.1 (0.9)	3
6	Completeness and sufficiency of meaning	2.1 (1)	4
7	Consistency and compliance of task structure	1.9 (0.6)	4
8	Discernibility (color tone and contrast)	1.9 (1.2)	4
9	Completeness and sufficiency of meaning	1.7 (0.9)	4
10	Completeness and sufficiency of meaning	1.5 (0.8)	4
11	Consistency and compliance of task structure	1.4 (1)	3
12	Discernibility (text size)	1.3 (0.75)	3
13	Button size (discernibility)	1.2 (0.9)	4

**Table 7:** Problems uncovered by end users and rated based on mean Likert scores.

Problem ID number	Heuristic category	Severity rating (0-4) (σ)	Max severity rating given (0-4)
1	Cognitive directness	1.83 (0.89)	3
2	Consistency and compliance of task structure	1.5 (0.7)	2
4	Discernibility (button size)	1.5 (0.8)	2
7	Discernibility (text size)	1.33 (1)	3
13	Discernibility (button size)	1.2 (0.9)	3
8	Discernibility (color tone and contrast)	1.15 (0.6)	2
9	Completeness and sufficiency of meaning	1(1.2)	3
6	Completeness and sufficiency of meaning	0.91 (0.6)	3
12	Discernibility (text size)	0.91 (0.7)	3

The older adult end user analysis found 14 problems, all of which were problems that had been identified by the expert group (the same problem ID number is used). Of the 13 problems listed in Table 6, 9 were uncovered by end users. These are presented in Table 7 in order of severity (as in Table 6).

Testimony from experts and users alike were used to provide insight into the problems and help designers better understand the problem. Themes were sought from the transcripts to uncover which characteristics of the interface experts and users most commonly found problematic. For example, regarding the login sequence for the smartphone app:

If not absolutely necessary this sequence should be removed from the use of the phone. At the very least it should be made sure that this only needs to be carried out by the clinician in the clinic once.

Maybe a voice password could be used or simply a pin number that only requires numerical values and does not require an email address.

Insufficient screen feedback and prompts for the user when carrying out certain tasks was identified as a recurring theme:

There should be a prompt to upload the data. When he (the user) presses the back button it should prompt the user that the data is about to be uploaded. The warning sign on the Exit pop-up box will cause anxiety and should be avoided.

I suggest that the interface should have one indicator saying if everything is working OK and if not, the interface should say specifically what the issue is.

The battery icon needs to change colour/shape when it is decreasing.; There needs to be a message which appears on the screen telling the user to initiate this (connection) sequence (PLEASE PRESS HERE TO ATTEMPT CONNECTION) and an indicator on the screen should tell them where to press.

Recommended by Expert 8

The size of screen elements such as icons, buttons and text were identified as being problematic:

(Made in reference to the pop up boxes in particular, for example “Invalid mail or Password” during login,) the screen needs to be utilised better, pop up boxes need to be bigger and more prominent.

There is no reason why the large screen space could not be utilised more effectively for these buttons (referring to exit pop-up buttons).

Expert 1

This (referring to an icon in top left hand corner to show that the app is running) is a good idea, but it is just too small for older adult users.

The results of the expert analysis and the end user analysis were compiled separately and then were presented in a problem report for system developers, with all problems listed with severity ratings and related testimony. The developers returned a proposal on how each problem could be solved, which were then reviewed by the usability engineering team. Examples of proposals that were accepted by the usability engineers are shown in Table 8.

Not all identified problems could be easily fixed by the system developers. Some aspects of the interface were built into the Android operating system (OS) and therefore could not be changed, whereas some problems could not be solved within the time constraints of the project. Where it was clear that the developer could not affectively address a problem through interface changes, the usability team proposed an alternative as to how the problem severity could be at least reduced if not completely eliminated. Some of examples of these problems are shown in Table 9.

**Table 8:** Problems that were directly addressed by system developers.

Problem ID number	System developer comments
2	The login will be a once off action carried out at the clinic to simply match the data coming through to the patient who is using the app. We have debugged the app so that any crashes should not mean the user has to log back into the app (login cookie is stored on phone cache). We will also make it so that the user can see the password as they are typing to decrease the chance of error, as suggested by the experts.
4	We will change this to a more prominent symbol that will be slightly bigger although is constrained by the operating system. We will make this symbol the same icon as the app icon.
6	We will change the feedback text to “Are you sure you want to close this application? After closing, the data will be sent to the server.” We will also change the caution symbol to an Information symbol based on your suggestion.
8	Contrast has been increased and text size increased to make it more prominent against the dark background.
9	We will remove the text “connect in 10 seconds pop-up” and just have “auto connection started” and “an everything is ok” pop-up once sequence is complete.
10	The “timer” text has been removed. We will also introduce colors for the symbols, red when the symbol is not in the ideal state, and green when it is.
11	We will introduce a green and red button choice with related symbols.
12	Text size will be increased and some redundant components will be removed from the interface to make more space.

**Table 9:** Problems that could not be directly addressed by system developers and which in turn had a proposed solution by the usability team.

Problem ID number	Problem	Scenario	System developer comments	Usability team proposal
1	The difference in operation between the home button and back button is not clear	User minimizes app	This is an Android design and cannot be changed and we feel that adding another button (an exit button) to the interface may cause further confusion	We will provide an instruction sheet that will show the user clearly the difference between the 2 buttons, emphasizing in particular that the back button is only used for uploading the data
3	Buttons on keypad are too small for this population	User logins to the app	This is an Android design and cannot be changed. The only solution would be to “buy” another keypad design that will be expensive	Short tutorials will be conducted for users on how to effectively use the keypad at the onset of use to improve confidence
5	Having to upload the data will be too hard to remember to do	Uploading data by exiting app	At this stage of development, an automatic data push is not feasible but will be considered for future	We will emphasize this scenario in our user manuals to reflect the fact that it needs to be carried out periodically
7	No prompt to indicate to the user that a manual connection is now required	User must connect to the insoles	We will improve the auto connection and introduce an option in the settings to turn off auto connection	We will describe the sequence in the short form manual, with steps for when a user should attempt a manual connection

#### Update of Paper Prototype and Development of First Working Prototype

**Figure 3:**
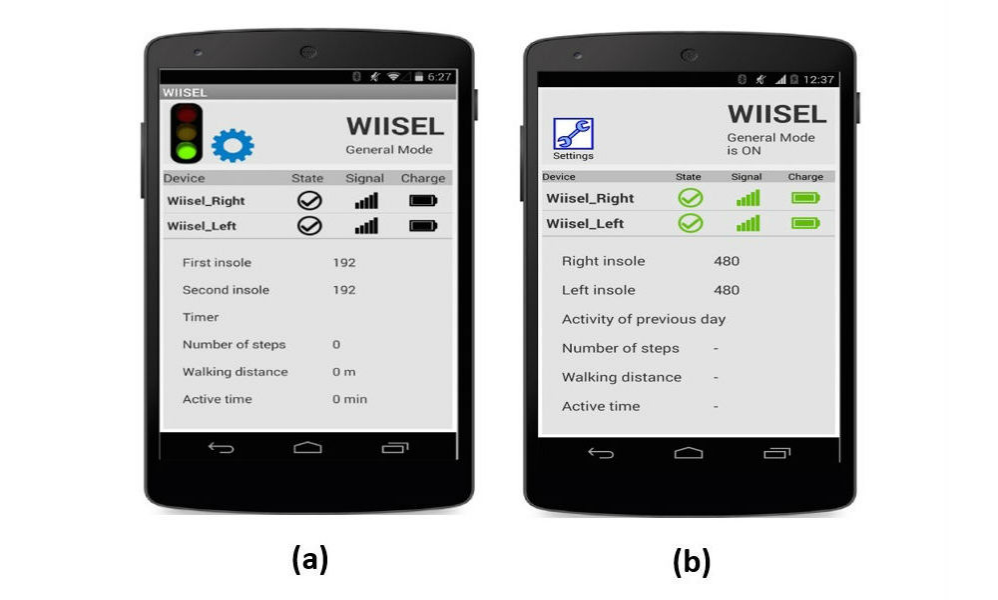
(a) The old interface showing the system status. Experts did not like the dull colors and crowded interface. Some users did not like the fact that there was no change of colors to indicate low battery, weak signal etc; (b) The updated interface with color indicators for connection, signal strength, and battery life, as well as increased text size and contrast.

**Figure 4:**
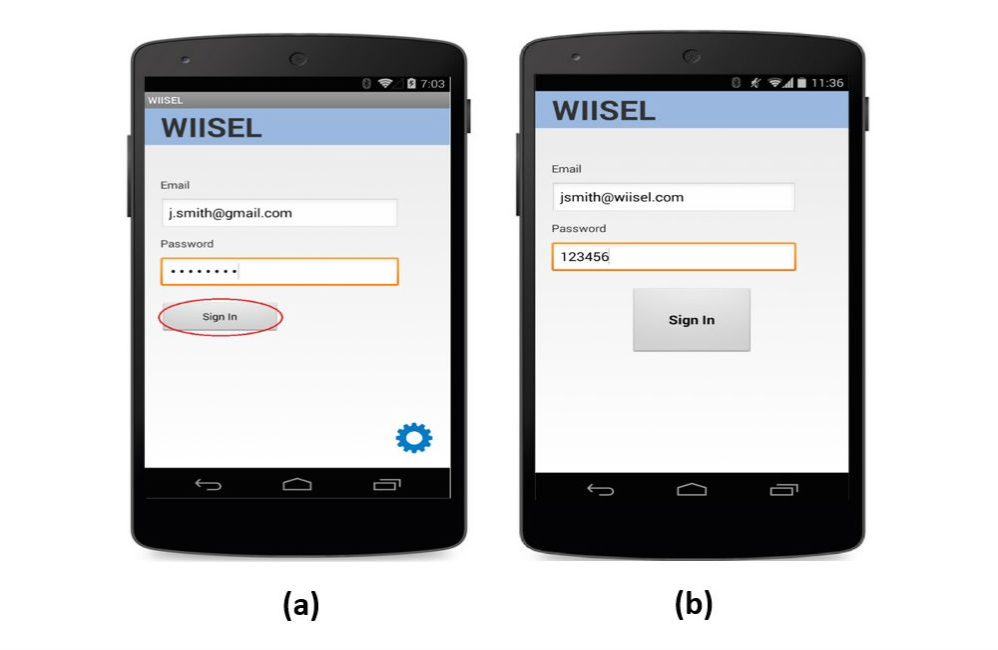
(a) Experts were concerned with the small button size and the fact that the password was encrypted meaning an older adult might lose their place when typing. This problem was also identified by end users; (b) Increased text size and a larger, more prominent sign in button as well as a decrypted password.

Based on this communication between the development team and the usability engineers, a working app prototype for the Google Nexus 5 smartphone was developed as well as a full set of user manuals based on the use cases and the feedback from the use case analyses. The use case was also updated to reflect the changes to the interfaces. Figures 3 and 4 show examples of how the updated interface (paper prototype version 2) compares with the paper prototype version 1. In Figure 3, we see how color indicators have been introduced to enhance the feedback on the system status screen. Text size has been increased and some elements have been removed from the interface to reduce crowding. Figure 4 shows how the login screen has been updated with a decrypted password as well as increased text size and button size.

**Figure 5:**
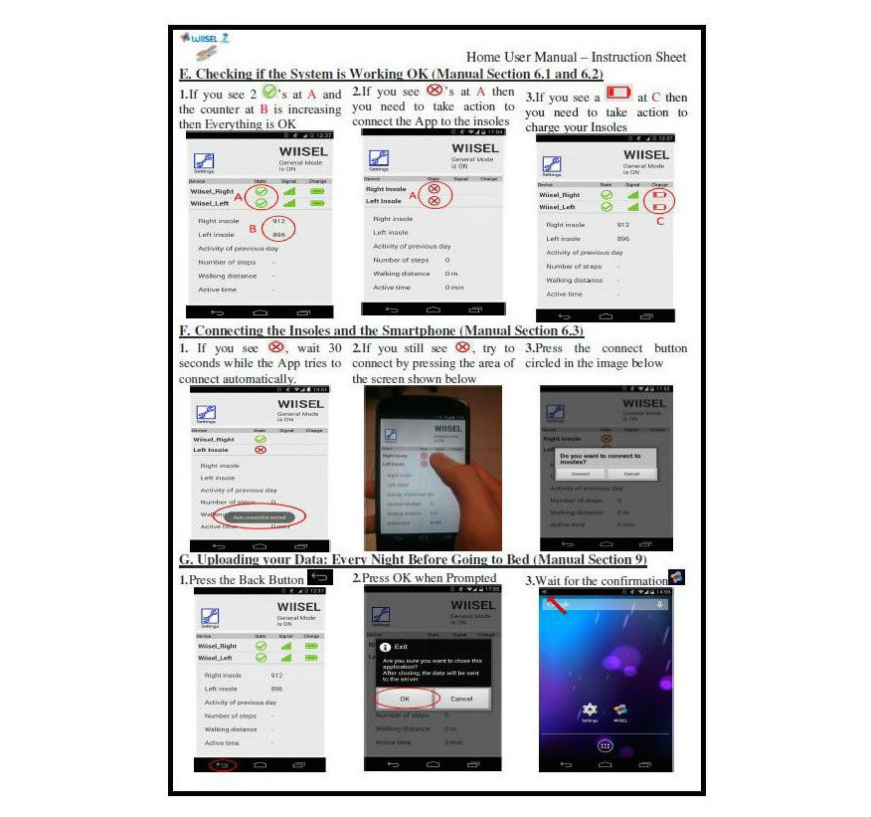
One side of the basic instruction sheet (short form manual) describing the connection and uploading sequences.

Where the problems identified by the experts could not be addressed by an interface change, user manuals were created to offset any confusion of difficulties the user might encounter with the interface. In order to create an effective user manual, the original use case was updated with all the interface changes made by designers. Each use case scenario now became a section of the user manual with the same chronological order maintained where applicable. For example, the use case scenario where the user connects to the insoles became a “how to connect” section in the user manual and was followed by a “how to upload” section, as in the use case. Two forms of manual were created, a short form manual entitled the “basic instruction sheet” which contained basic instructions on a double-sided laminated sheet, and a longer form manual laid out in similar style to the use case that elaborated on the instructions provided in the basic instruction sheet and provided additional instructions for procedures that would not be considered routine. Another version of these 2 forms were also created for clinicians with additional information on how to set up the system for the user, change settings, calibrate insoles, and adjust fall detection settings. A selected sections of the manual is presented in Figure 5.

### Phase 2: Expert Inspection Results

#### Use Case Inspection of Paper Prototype Version 2

Table 10 presents examples of how the various problems uncovered during the use case analysis in phase 1 were addressed and compares the problem rating it received from the first use case analysis (paper prototype version 1) with the new rating it received from the analysis of the updated interface in phase 2 (paper prototype version 2).

The inspection found that of the 21 original problems identified by the experts, 3 had now received a rating of 0 from the experts, 17 had received decreased ratings, and 1 (ID# 11) had received an increased rating.

**Table 10:** Comparison of problem ratings between paper prototype V1 problems and the updated interface (paper prototype V2). The max individual score that was given by the 10 experts is also included to highlight the fact that some experts may have given a more severe rating than the mean or standard deviation indicates.

Problem ID number	Severity rating (0-4) (σ)	Max severity rating given (0-4)	How was the problem addressed?	Severity rating (0-4) (σ)	Max severity rating given (0-4)
	Paper prototype version 1			Paper prototype version 2	
1	2.5	4	A manual section was added that explained the operation of each button in the context of overall phone operation and in the context of the WIISEL^a^app.	1.4	2
2	2.4	4	Debugging of the app and improved connection sequence means that app resets are not as likely, leading to a decreased need for the user to have to login. Button size was increased and the password decryption during the sequence was removed.	0.3	1
5	2.1	3	Additional manual information was added and instructions on setting a daily reminder on the phone.	1.4	2
6	2.1	4	The caution symbol has been replaced with an information symbol, additional text information has been added explaining to the user what is happening regarding the data upload.	0.4	1
11	1.5	3	Red and green have been introduced as “I have fallen” option (red) and “I am Ok” option (green). Whereas experts agree with the notion of illustrations and color coding, they are now concerned that there is no text labels on the buttons. One expert pointed out that red could be confused for a cancel button (ie, to cancel the alarm) in the same way as it would be when answering a phone call. This could lead to a user accidentally sending a fall alert to carer during a false positive sequence in which the user is forced to press a button in a hurry.	2.1	4
10	1.5	4	The addition of the green indicators for “good” and orange and red indicators for “bad” such as for the battery symbol have been welcomed.	0.1	1
12	1.3	3	Whereas the homescreen interface had improved, some experts felt that some space was not being utilized well and that small text and crowding was still an issue.	0.44	1

^a^WIISEL: wireless insole for independent and safe elderly living.

**Table 11:** Average metrics and consensus for 9 experts. After scenario questionnaire (ASQ) scores range from 1-7, where 1 is the most satisfied and 7 is the least satisfied the user can be.

Scenario	Time taken (s)	Average errors made	ASQ^a^ score	Comments
Check the system status	4.7	0.5	2	The increasing numbers (referring to the incrementing counters) on the interface are still unclear to some experts. Whereas the experts acknowledge that this indicates “data is streaming,” the indication should be that it is either connected or it is not, any other information than that is completely redundant. Documentation is a little crowded, would like to see more space given in the manual
Connect to the insoles	48.0	0	3	This task but could be made easier by giving more feedback to the user and simplifying the interface somewhat. If the connection takes a couple of minutes then the user needs to be made aware that something is happening or else they will just keep pressing the connect button, possibly causing a crash or accidentally disconnecting it. The ambiguities in the connection sequence need to be made clear in the manuals, ‘’don’t panic, give the system a chance etc.’’
Upload data	4.3	0	2	There is a concern that there is no immediate feedback to let the user know they have completed the task successfully. The manual indicates that an icon will appear in the top left hand corner of the screen, however, it is obvious that this does not appear straight away if there is a lot of data, this should be made clear in the manual or just removed, as it may cause anxiety.
User minimizes app			4	The difference in operation between the back button and the home button, while addressed, is not made completely clear in the user manual. This will be important for users particularly if they intend to user other functions on the phone.
Reset app	23.4	0.7	3	Not a very straight-forward sequence given the number of screens that need to be navigated, but under supervision this should be OK. This is quick if the user knows what they are looking for, although they could get easily lost. The user manual should explain to the user that they made need to scroll down in each menu to reach the option they need. If the user does not see the exact same screen that they see in the user manual they will think something is wrong.
Login to app	27.0	1.1	3	This will present challenges, particularly the keyboard. If the user can follow the manual then it will be easy but any digression from the main path will cause problems. The time is OK, although mistakes with the user credentials will obviously increase the time as well as the user frustration. More steps need to be added to this sequence in the manual.
Respond to fall alarm	7	0.3	3	This is an easy sequence but the confusion over the options makes it a little bit more burdensome especially on users with any form of cognitive impairment. Very quick to do, provided the user is clear on what option they are pressing. The documentation here is inadequate and needs to explain the situations in which each option may need to be pressed.

^a^ASQ: after scenario questionnaire.

#### Expert Cognitive or Contextual Walkthrough With Working Prototype Version 1

Table 11 shows the captured average metrics from each scenario, with the time and errors made metric captured. Accompanying the metrics are a selection of comments from experts.

**Figure 6:**
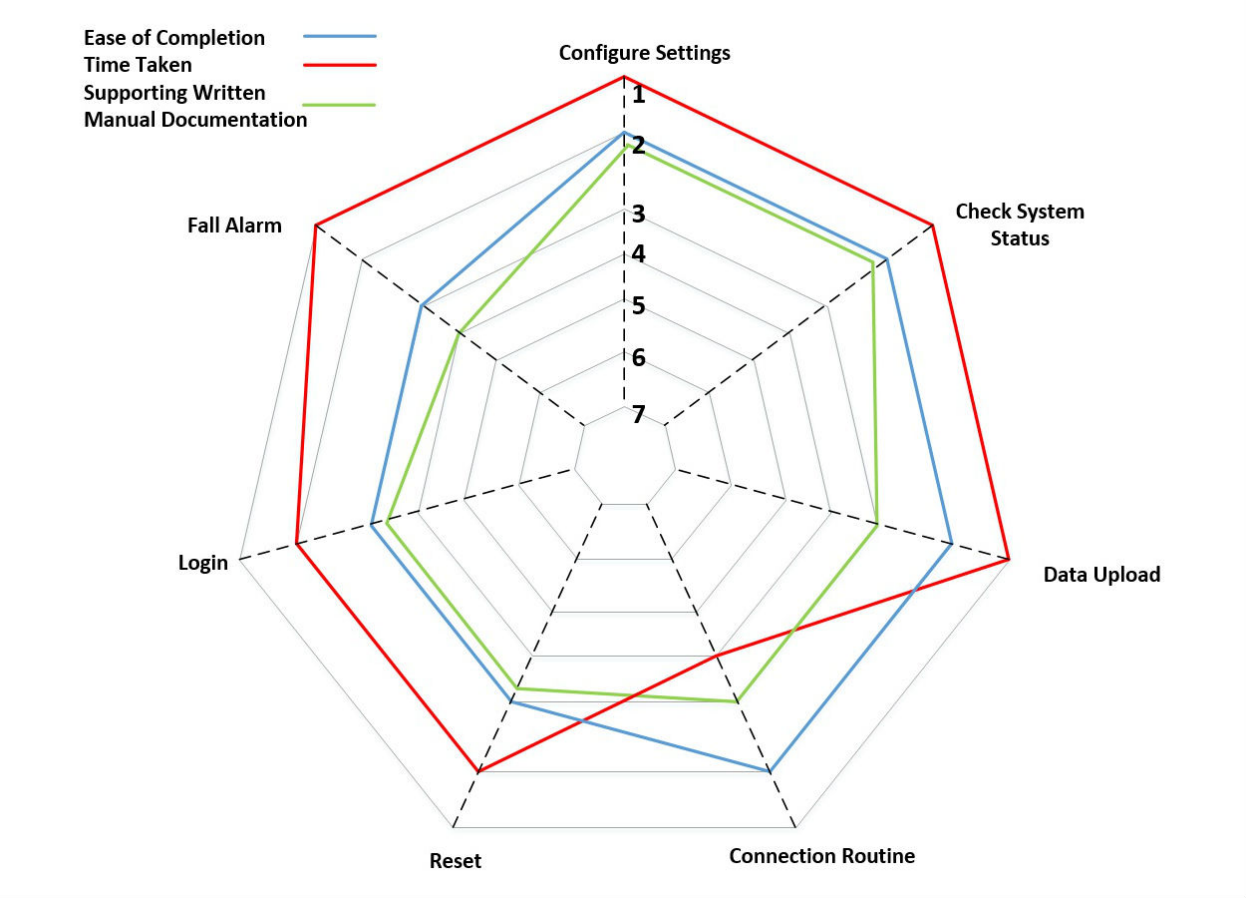
All basic scenarios scored consistently well regarding ease of completion (blue) with just slight superficial changes, the more challenging scenarios such as login and reset registered higher (worse) scores. Only one scenario, connection routine, scored poorly in the time taken (red) metric, owing to the length of time it takes the insoles to sync with the app. Several experts were confused by some of the layout and instructions in the manuals (green), with improvement required for several scenarios, particularly the instructions for the fall alarm sequence.

Of the 8 scenarios, three achieved a score of “satisfied”and four achieved a score of “somewhat satisfied,” whereas one achieved a neutral score. No scenarios scored a perfect score of 1, indicating that all scenarios require some improvement, particularly regarding the clarity and flow of the supporting documentation. These data are best illustrated in a radar chart (Figure 6). A radar chart allows for multiple data series to be displayed across common variables, each variable having its own axis (the dotted line). The axis values go from low to high as you read toward the center of the chart, with lower scores indicating a better outcome (data points near the edge of the chart). The chart in Figure 6 shows how the 3 individual components of the ASQ score, satisfaction with ease of completion, time taken, and effect of supporting documentation.

In response to comments by the experts during the inspection, the user manuals were updated, and several minor changes were made to the interface. These updates are listed in Table 12.

**Table 12:** Changes made to the user manuals and interface based on expert inspection.

Scenario	Suggestions	Changes made
Check the system status	This section of the manual needs to be less crowded	The documentation now includes 6 steps instead of the original 4. A step is included to explain that the app may take several seconds to start up and how to lock the phone again. Increased text size
Connect to the insoles	Would like to see some explanation of the crash sequence in the user manual.	The same number of steps is maintained with additional labels indicating where on the screen the user may have to press during the connection sequence. Increased button or text size
Upload data	Manual indicates that an icon will appear in the top left hand corner of the screen, however, it is obvious that this does not appear straight away if there is a lot of data, this should be made clear in the manual or just removed, as it may cause anxiety.	The third step, which explained that an icon would appear upon successful completion has been removed to avoid confusion as it does not always appear straight away. The section now also includes further explanation of what the back button is used for. Increased button or text size
User minimizes app (home button)	The difference in operation between the back button and the home button, while addressed, is not made completely clear in the user manual.	A section explaining the function of this button was placed on the same page as the section explaining the use of the back button. This was done in order to provide a clear distinction between the function of the 2 buttons
Reset app	The user manual should explain to the user that they made need to scroll down in each menu to reach the option they need. If the user does not see the exact same screen that they see in the user manual, they will think something is wrong.	Expanded from a 3-step instructional process to a 5-step process. A section was also introduced to explain to the user how to best interact with the touchscreen in terms of scrolling and striking
Login to app	More steps need to be added to this sequence in the manual	Expanded from a 4-step process to a 6-step process including additional instructions on how to access the number keypad and find the @ symbol Increased button size
Respond to fall alarm	The documentation here is inadequate and needs to explain the situations in which each option may need to be pressed. As regards the options, it is suggested that red and green not be used to distinguish options and that text labels also be used for the buttons to accompany and supplement images	Expanded from 1-step to a 3-step process with clear illustrations to show when the user might experience New buttons introduced to indicate cancel and confirm Increased button size

**Figure 7:**
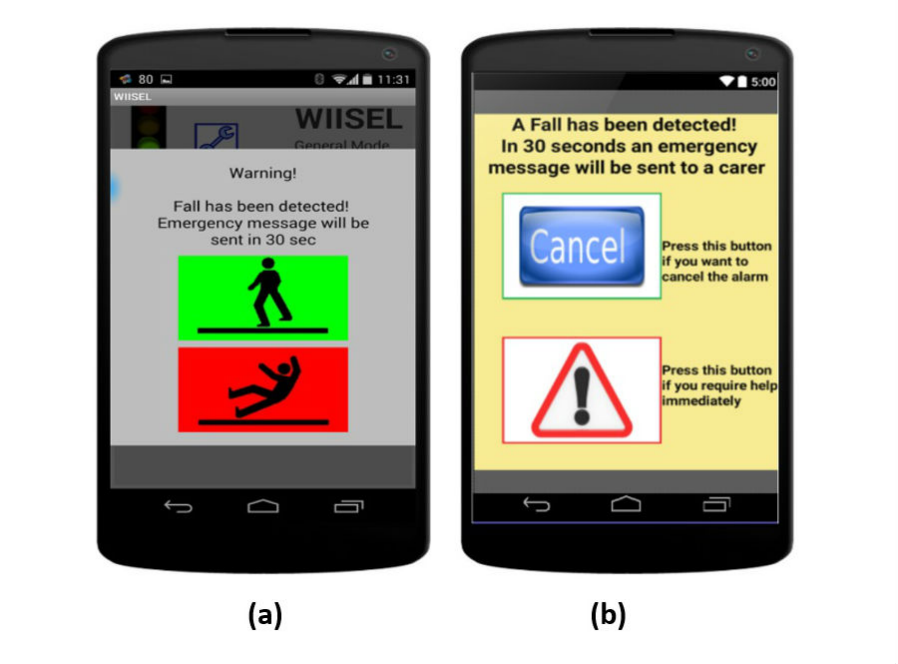
(a) Fall alarm interface before expert inspection, the red and green caused confusion as the red was associated with “cancel” as you would find on a phone call interface; (b) Fall alarm interface after expert inspection, a more appropriate symbol was introduced for the help button whereas the cancel button was changed to a more neutral blue with appropriate labeling.

These changes led to working prototype version 2 and a new set of user manuals that now contained 4 laminated sheets. Figure 7 shows an example of how the fall alarm interface has been updated.

### Phase 3: Usability Testing With End Users

**Figure 8:**
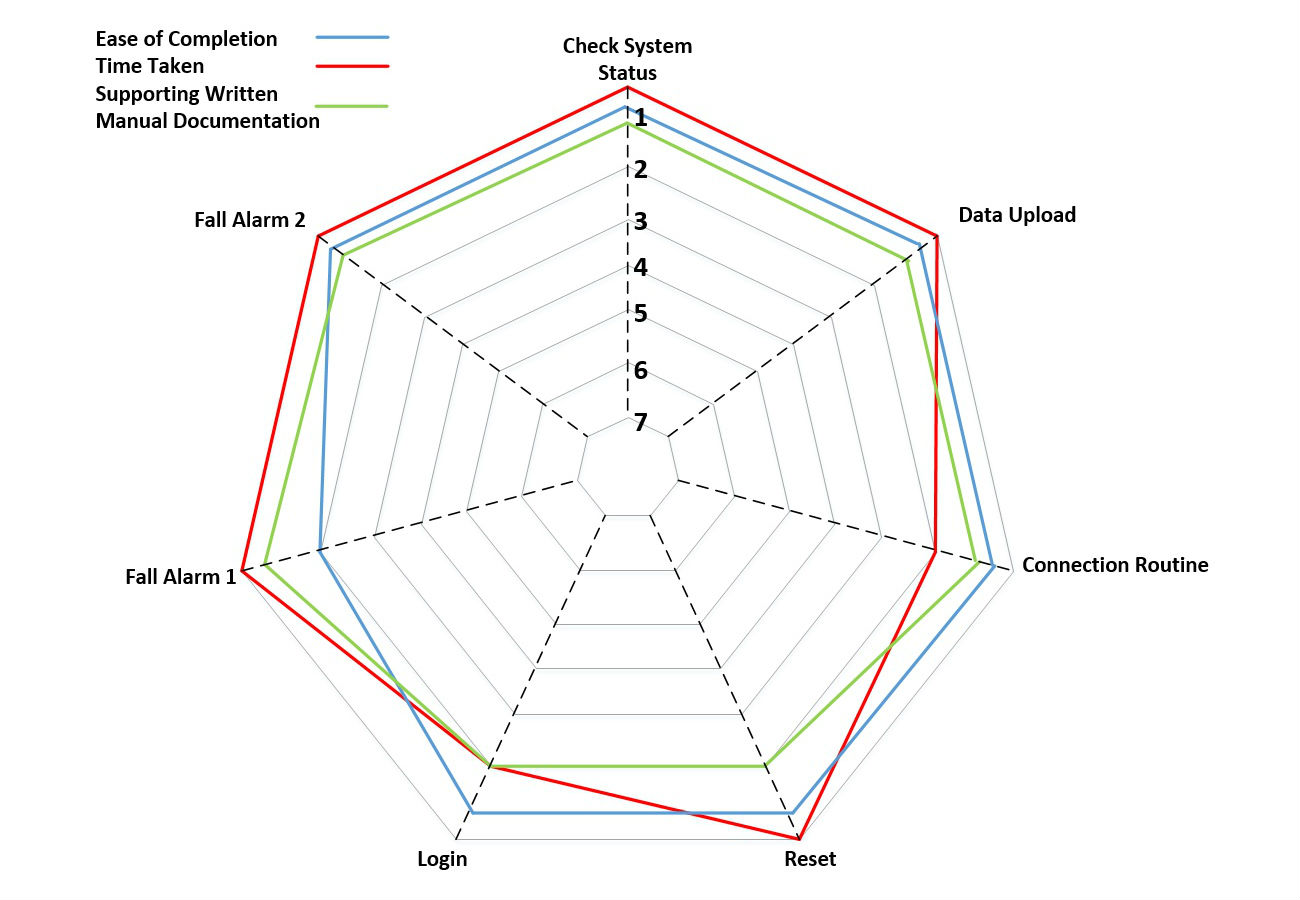
All scenarios scored maximum for ease of completion (blue) apart from the fall alarm 1 which caused slight confusion. Time taken (red) was not considered a major issue for any of the scenarios, with the connection routine not scoring maximum due to the nature of the syncing process, whereas the unfamiliarity with typing caused some users to mark down the login sequence. There was some confusion with the reset and login sequences in the user manual.

Table 13 shows the average metrics for the 10 test participants during the usability testing of working prototype version 2, whereas Figure 8 illustrates the breakdown of the ASQ metric in terms of satisfaction with ease of completion, time taken, and support documentation (there was some confusion with the reset and login sequences in the user manual (green) which is explained further in Table 13).

The results of the NASA-TLX was performed on paper and the metrics are shown in Table 14. A score of 100 indicates maximum burden on the user, whereas a score of 0 indicates no burden. The first 4 tasks scored very well, indicating little to no burden on the user. The login and reset procedures, due to the number of steps involved, created the most mental, physical, and effort burden, as well as the most frustration, particularly the login procedure. The most temporal burden was created by the fall alarm procedure, due to the timer on the screen, forcing the user to make a hasty choice.

**Table 13:** Performance metrics for each scenario during user testing with working prototype 2, with related commentary as observed during the testing. The after scenario questionnaire (ASQ) score ranges from 1-7, where 1=best score possible and 7=worst score possible.

Scenario	Time taken	Errors made	ASQ^a^	Comments
Check the system status	19	0.4	1	All users found this very easy to complete and manuals clear to follow. The only errors encountered were when users released the screen slide lock too early, which occurred with 4 of the 10 users.
Connect to the insoles	31	0.13	1	Whereas users found the procedure and manual easy to follow, the time taken for the sync to complete caused minor frustration. The only error encountered were when some users held the manual connection button for too long.
Upload data	13	0.13	1	All users found this very easy to complete and manuals clear to follow. Some minor errors included pressing the cancel button instead of the OK button. Whereas the OK button was clearly marked as the button to press in the user manual, sometimes the user would press cancel without consulting the manual.
Reset app	112	1.0	2	While quite a complex sequence, most user’s found it easy to complete, but were susceptible to minor errors, such as accidentally pressing the wrong menu option, or accidentally pressing while scrolling. These errors are down to unfamiliarity with touch screen interfaces and “heavy handedness.” There was one error with regards to the layout of the manual.
Login to app	171	0.88	2	This sequence took the most time, due to most user’s unfamiliarity with touchscreen keypads. There was a huge disparity in times, ranging from 30s to nearly 5 min, with those who had previous experience with smartphones faring generally better. The manual layout also caused some confusion with user’s having to jump a step to find out how to enter numbers and then having to return to the previous step.
Respond to fall alarm	6	0.5	2	The original fall sequence caused an error for every second user, who thought the red option was the cancel option, as you would expect on a mobile phone call.
Respond to fall alarm	6	0	1	The new alternative fall sequence proved more successful, with the removal of the red or green option causing less confusion with no errors reported.

**Table 14:** NASA Task Load Index (NASA-TLX) scale breakdown by scenario. The NASA-TLX score ranges from 1-100, where 1=worst score possible and 100=best score possible.

Scenario	Overall score	Mental	Physical	Temporal	Performance	Effort	Frustration
Check the system status	4.9	3.8	8.5	4.3	4.3	4.7	4.0
Connect to the insoles	7.0	9.9	5.6	12.9	4.4	4.4	4.9
Upload data	4.1	3.2	4.0	3.3	5.3	3.3	5.2
Minimize app	3.6	4.7	3.7	2.8	3.0	3.8	3.5
Reset app	30.3	42.3	16.3	15.0	17.5	53.3	37.2
Login to app	39.1	54.7	29.0	20.3	20.7	65.8	43.8
Respond to fall alarm 1	26.6	43.5	7.7	59.5	20.2	18.3	10.5
Respond to fall alarm 2	13.6	22.8	6.2	33.3	4.8	6.5	7.7
Most burdensome scenario	Login	Login	Login	Respond to fall alarm 1	Login	Login	Login

**Table 15:** Likert items severity rating (range 0-4, 0=no problem, 4=most severe problem) for interface ergonomics by scenario. Some Likert items did not apply to certain scenarios. An x indicates that there was no Likert statement for that particular interface aspect for that scenario.

Scenario	Color	Text	Buttons	Keypad buttons	Icons size	Icon meaning
Check the system status	0	0.25	x	x	0.12	0
Connect to the insoles	x	x	0.12	x	x	x
Upload data	0	0.12	0.12	x	x	x
Login to app	0	0.12	0	0.37	x	x
Respond to fall alarm 1	0	0	0	x	x	x
Respond to fall alarm 2	0	0	0	x	x	x

**Table 16:** Presents the evolution of three distinct problems through the testing lifecycle with the usability metrics taken at each stage.

Problem ID number	Phase 1	Phase 2		Phase 3			
	Severity rating (0-4) (σ)	New severity rating after inspection	Expert ASQ^a^ score (0-7)	End user ASQ score (0-7)		NASA-TLX (0-100)	Caused error during user testing?
2	1.77	0.6	3	2		39	On average, users made 0.88 errors during this scenario
6	1.41	0.4	3	1		4	On average, users made 0.13 errors during this scenario
11	1.09	1.2	3	1		13	On average users made 0 errors during this scenario

^a^ASQ: after scenario questionnaire.

**Table 17:** System usability scale (SUS) metric, split into overall usability and learnability, captured at each phase.

	Phase 1		Phase 2		Phase 3		
	Learnability x̄ (σ)	SUS^a^ total x̄ (σ)	Learnability x̄ (σ)	SUS total x̄ (σ)		Learnability x̄ (σ)	SUS total x̄ (σ)
Experts	35 (24.23)	55 (23.6)	48.75 (26.36)	68.75 (11.6)		n/a	n/a
End users	58 (26.43)	78 (10.77)	n/a	n/a		87.5 (5)	88 (3.75)

^a^SUS: system usability scale.

Table 15 shows the Likert response for different aspects of the interface in each scenario. The severity rating is calculated in the same manner as phase 1 and 2.

### Summary of Results

With combined expert and end-user analysis of a comprehensive use case having originally identified 21 problems with the system interface, we have only seen observed 3 of these problems in user testing (problem ID 1, 2, and 12). Satisfactory ASQ ratings were obtained during validation testing by both experts and end users, and final testing by users shows the system requires low mental, physical, and temporal demands according to the NASA-TLX. Table 16 shows how three of the problems (problems involving flow, consistency, and feedback) have evolved over the testing cycle. Problem 2 and 6 show a clear linear improvement from phase 1-3, with problem 2 an example of a problem that despite best efforts remained a cause of potential user frustration due to the unfamiliar style of touchscreen keyboards. Problem 6 represents an example of a problem that was effectively mitigated through interface changes and manual support. Problem 11 is an example of a problem that was actually exasperated by an interface change, causing greater confusion to users, although this was effectively identified and mitigated between phase 2 and 3.

The system usability scale (SUS) metrics after each phase are presented in Table 17. The SUS is split into 2 scales: (1) overall usability and (2) learnability [29]. Early phases showed widely variable SUS scores, particularly among experts, whereas phase 3 scores showed agreement among end users that the interface had achieved some level of acceptability.

## Discussion

### Overview

We have presented a multi-phase, mixed-method HCD approach to improve the user experience of a smartphone interface, which forms part of a connected health system. Our approach was designed to uncover and mitigate any usability problems as early as possible, before they were exposed to end users during usability testing and in formal clinical trials. This paper presents one full cycle of our HCD process, with each phase representing an iteration where a design update or refinement took place. Our approach has met the specific recommendations for a HCD process [30]. We have adopted the input of multi-disciplinary skills and perspectives by eliciting the feedback of both an end-user group and an appropriately experienced expert group throughout the process. We have sought to gain an explicit understanding of users, tasks, and environments and consideration of the whole user experience through the adoption of a use case that provided context of use for system tasks and scenarios and through the examination of the perceptual and cognitive needs of the target end user. We utilized a user-centered evaluation driven design using standard usability evaluation metrics at each point in the cycle. We involved users throughout the design process, at both early and later stages. Finally, we employed an iterative process, split into 3 stages or phases that allowed for user feedback to be worked into design updates.

### Principal Findings

From our observation of older adults’ interactions with smartphone interfaces, there were some recurring themes. Clear and relevant feedback as the user attempts to complete a task is critical (in line with contemporary literature) [31,32]. Feedback should include pop-ups, sound tones, color or texture changes or icon changes to indicate that a function has been completed successfully, such as for the connection sequence (problem ID# 9). For text feedback, clear and unambiguous language should be used so as not to create anxiety, particularly when it comes to saving data such as in the data upload sequence (problem ID# 6). Older adults not familiar with technology are often afraid that they might delete something by accident or fail to save important data properly. Warning tones or symbols, such as a caution symbol, should only be used if absolutely necessary. For audio feedback, clear and low frequency tones should be used. Login sequences where the user is required to input text with a QWERTY keyboard should be avoided (problem ID 2), particularly for those who have no previous touchscreen experience. If a login sequence is considered necessary for security or identification purposes, it should be ensured that a login process is made as simple as possible (do not hide password, be clear about what username is required, supply ample support documentation for process). For simple interface elements, text sizes should be at least 10pts (Didot system), whereas button sizes should have a surface area of no less than approximately 200mm^2^ [11,33].

In terms of metrics, we used 4 different subjective measurement systems (Likert scales, ASQ, NASA-TLX, and SUS) to assess the usability of the interface at different stages. The Likert scales allowed for quick satisfaction ratings of the perceived ease of use of each task in the use case and of the suitability of interface elements such as text and button size. The ASQ was more suitable for postscenario ratings when the user had actually completed the task, whereas the NASA-TLX was used to supplement the ASQ to provide further details on what kind of burden, be it physical or cognitive, the task placed on the user. The SUS was utilized when the user had completed a full use of the system and carried out all tasks. We observed that all of these metrics are providing the similar information, just in slightly different resolutions, and that a mixture of metrics allows us different insights into user perceptions of usability. For example, in phase 3, from looking at the ASQ scores of the login sequence, we could conclude that the user was satisfied with the ease of the task. However, when we looked at the NASA-TLX scores, we observed that the task was creating a large mental demand on them. These 2 metrics, whereas showing us seemingly conflicting pieces of information, may be telling us that the user judged the task as being easy simply because they completed it successfully, regardless of the difficulty they encountered or the time it had taken them. It is only when they think about the task in terms of the NASA metrics that they become honest about what kind of burden the task placed on them. The SUS was a useful general indicator of overall usability but its wide variability (Table 17) suggests that it is best used with larger sample sizes. High SUS scores do not guarantee the system will not suffer usability problems in the field [34]. These metrics are probably best used to supplement more objective metrics such as task times and error rates.

### Procedural Observations

In terms of efficiency, our methodology proved to be successful. The utilization of the use case analysis activities during phase 1 provided a focus for all stakeholders on the context of and the intended use of the system. The time it took for each individual to analyze and provide feedback was on average 1 h. Within this hour, the individual was experiencing and commenting on context, was being formally interviewed, was filling out questionnaires, and was providing opinions on interface concepts. Therefore in one session the use case analysis provides multiple streams of data, whereas in previous literature, this kind of feedback would need to be gathered across multiple activities, such as surveys, interviews, and ethnographic observations. In phase 2, the use of expert inspection groups also proved highly efficient. We recommend that research groups and design teams maintain an inspection group who can carry out on hand inspections of new system versions. This group, which can comprise 4-6 members, need not necessarily be qualified usability engineers but can be trained in techniques such as heuristic evaluations and cognitive walkthroughs. In terms of how long it took to complete each phase, as this was a case study as part of a research project, the amount of time spent on each phase was probably drawn out longer than it would be in a more industrial setting. In all, the 3 phases together took approximately 12 months, with phase 1 taking the bulk of the time (approximately 6 months) as use cases were developed and redeveloped and end users were interviewed and tested. After the app was developed and testable, the phases became shorter, with phase 2 and 3 taking approximately 3-4 months each. As the methodology is applied in future, it will become more refined, allowing for quicker development cycles.

### Limitations

Time and technology constraints meant that not all design requirements could be implemented. For example, the replacement of the manual data upload with an automatic periodic data upload could not be implemented in time by the engineering team. Similarly, the structure of the Android OS meant that some user and expert recommendations could not be implemented, particularly regarding the positioning of pop-ups or the nature of data storage. Some design changes led to a decrease in user experience, particularly for the fall alarm sequence (problem ID# 11). It became clear during user testing that the use of red and green in an emergency situation may not be the best practice, with some users confusing the red emergency button for a cancel button, like it may be presented on a phone call screen (red for “hang-up”). In this case, the design team failed to take into account the recommendation of one expert who predicted that a red or green option may cause confusion. We can conclude from this that taking on board opinions from different stakeholders can present a challenge for designers. However, the nature of our iterative methodology meant that this problem was identified and addressed between phase 2 and 3.

In phase 1, the older adult end users tended to be very optimistic about how they would handle the system and the smartphone interface, overall giving higher scores in response to Likert statements and for the overall SUS score. Experts tended to be more pessimistic but this was probably due to their vast experience with older adults and technology. Most experts conceded that the use case analysis was a hypothetical one and that the capabilities of the older adult population are extremely variable, however, they felt that it was an extremely useful exercise in identifying major potential problems and addressing them early in the design process. Despite the difference in outlook between the experts and older adults, both groups reached agreement on most problems, particularly about the perceived difficulty of the login process and the lack of clear feedback when checking the system status and during the data upload process. We can conclude from this that utilizing multiple perspectives from different groups is an important feature of a good human-centered design process.

### Conclusions

The HCD Methodology we have designed and implemented based on the principles of ISO 9241-210 has produced a functional app interface that is now suitable for exposure to older adults in long term clinical trials. We have applied appropriate testing techniques given the context of the interface being assessed. We would consider this a thorough and robust method for testing and informing design changes of all types of interactive connected health systems.

## Abbreviations

ADL: acitivities of daily living
ASQ: after scenario questionnaire
CD: color discrimination
CS: color sensitivity
GP: general practitioner
HCA: high contrast acuity.
HCD: human-centered design
HCI: human-computer interation
HRB: health research board
IT: information technology
LCA: low contrast acuity
LCALL: low contrast acuity in low luminance
OS: operating system
RA: reading acuity
SUS: system usability scale
UHG: University Hospital Galway
